# Self-Healing Cellulose Nanocrystals-Containing Gels via Reshuffling of Thiuram Disulfide Bonds

**DOI:** 10.3390/polym10121392

**Published:** 2018-12-15

**Authors:** Wenyan Li, Shengchang Lu, Mengchan Zhao, Xinxing Lin, Min Zhang, He Xiao, Kai Liu, Liulian Huang, Lihui Chen, Xinhua Ouyang, Yonghao Ni, Hui Wu

**Affiliations:** 1 College of Material Engineering, Fujian Agriculture and Forestry University, No. 63, Xiyuangong Road, Fuzhou 350108, China; 1161033002@fafu.edu.cn (W.L.); 1141033002@fafu.edu.cn (S.L.); zmc0622@126.com (M.Z.); fafuxin@163.com (X.L.); mzhang@fafu.edu.cn (M.Z.); xiaohe_river@163.com (H.X.); liuk1103@163.com (K.L.); hll65212@163.com (L.H.); fafuclh@163.com (L.C.); 2 Department of Chemical Engineering, Limerick Pulp and Paper Centre, University of New Brunswick, Fredericton, NB E3B 5A3, Canada

**Keywords:** cellulose, disulfide, gel, self-healing

## Abstract

Self-healing gels based on reshuffling disulfide bonds have attracted great attention due to their ability to restore structure and mechanical properties after damage. In this work, self-healing gels with different cellulose nanocrystals (CNC) contents were prepared by embedding the thiuram disulfide bonds into gels via polyaddition. By the reshuffling of thiuram disulfide bonds, the CNC-containing gels repair the crack and recover mechanical properties rapidly under visible light in air. The thiuram disulfide-functionalized gels with a CNC content of 2.2% are highly stretchable and can be stretched approximately 42.6 times of their original length. Our results provide useful approaches for the preparation of dynamic CNC-containing gels with implications in many related engineering applications.

## 1. Introduction

Self-healing materials have received significant attention because of their ability to restore structure and mechanical properties after damage, which can be applied to various applications such as coatings/sealants, tissue adhesives, and drug/cell delivery [1,2,3,4,5]. Healing agents including cross-linking reactants and catalysts were applied in self-healing systems initially [6]. Upon mechanical damage, these agents, in the encapsulation of nanotubes and microcapsules, were released and subsequently polymerized within the crack so that the damages were fixed [6,7]. Recently, materials using reversible chemical bonds to repair damages in polymeric materials were explored extensively [1,2,3,4,5]. Generally, non-covalent interactions and dynamic covalent bonds were employed in the creation of reversible self-healing systems. The non-covalent bonds usually include coordination interactions [8,9,10], hydrogen bonds [11,12,13], hydrophobic interactions [14], electrostatic interactions [15], host–guest interactions [16], and π–π stacking [17]. Metallosupramolecular polymers comprising the hard phase of metal–ligand complexes and soft domains of the hydrophobic core were fabricated [8]. The cracks can be healed by photothermal conversion. An autonomic self-healing material with high stretchability was synthesized by the introduction of a Fe(III)-2,6-pyridinedicarboxamide coordination complex in a poly(dimethylsiloxane) matrix [9]. Thermoplastic elastomers prepared using polystyrene backbone as hard phase and polyacrylate amide brushes as soft phase were healable through hydrogen bonding [11]. A pressure- and flexion-responsive material obtained by embedding nickel nanostructured microparticles into supramolecular organic polymer can heal the crack via hydrogen bonds [12]. Although the non-covalent interactions offer intensive reactivity and repeatability of the repair reaction, the gels composed of physical crosslinks are weaker [18].

In contrast, reversible covalent bonds, such as disulfide bonds [2], imine bonds [19], acylhydrazone bonds [20], and didiol−borax complexations [21], provide relatively stronger molecular interactions and strength for self-healing materials. Under external stimuli such as heat, light, and pH, the dynamic reactions allow the breakage and re-forming of bonds to achieve repair. In particular, disulfide chemistry, one crucial part of dynamic covalent chemistry, is unique in the controllability of exchange reactions [22,23,24,25,26,27,28,29,30,31]. A self-healing copolymer with dual disulfide bonds and supramolecular crosslinked network was prepared [22]. The samples restore its original shape and stretch to two times after the two separated pieces contacted tightly for 24 h. By introducing the cyclic disulfide, the reactivity of disulfide bonds enhances, and the hydrogel heals under neutral/mildly acidic conditions [23]. Self-healing polymers were fabricated by the reshuffling of trithiocarbonate units under UV irradiation [24] and the reforming of disulfide bonds under visible light [25]. However, the introduction of non-environmentally friendly chemicals and complex reaction condition are usually requested to construct self-healing systems, which is not practical for the environment and mass production.

Cellulose is an abundant green resource in nature. Owing to the high intensity of hydroxyl groups along the skeleton of cellulose, cellulose is allowed to generate functional materials via physical and chemical modification [32,33,34,35,36,37,38,39,40,41,42,43,44,45,46,47]. In particular, the cellulose nanocrystals (CNC) have attracted a tremendous amount of interest owing to its fascinating physicochemical properties such as adaptable surface chemistry and high mechanical strength. Nanocellulose was widely used as a reinforcing agent to enhance the mechanical properties of materials, which have potential applications in nanocomposites, electronics, membranes, and supercapacitors [48]. However, reports on CNC-containing gels with rapid self-healing and high stretchable properties are still rare. Therefore, it is highly desired to explore natural resource-derived gels incorporating disulfide bonds with rapid self-healing and high stretchability using CNC as building blocks.

Herein, we report a CNC-containing gel by incorporating dynamic disulfide covalent bonds prepared via polyaddition. The CNC-containing disulfide-functionalized gels with high stretchability are capable of self-healing and recovering the mechanical properties under visible light rapidly.

## 2. Experiments

### 2.1. Materials

Bamboo dissolving pulp was purchased from Sichuan Tianzhu Resources Development Co., Ltd. (Yibin, Sichuan, China). Hexamethylene diisocyanate (HDI), triethanolamine (TEA), *N*,*N*-dimethylformamide (DMF), ditin butyl dilaurate (DBD), carbon disulfide (CS_2_), iodine (I_2_), tris(2-carboxyethyl)phosphine hydrochloride (TCEP) were purchased from Aladdin Industrial Corporation (Shanghai, China). 2-(ethylamino)ethanol was obtained from TCI (Shanghai) Development Co., Ltd. (Shanghai, China). Chloroform (CHCl_3_) was supplied by Sinopharm Group Chemical Reagent Co., Ltd. (Tianjin, China).

### 2.2. Preparation of Cellulose Nanocrystals (CNC)

CNC were prepared from bamboo pulp by hydrolysis with sulfuric acid. Bamboo pulp was immersed in water and stirred for 1 day. The concentrated sulfuric acid (95%) was added dropwise into the mixture under ice bath until the 64% acid concentration was reached. After the suspension was stirred at 45 °C for 3 h, the mixture was diluted with deionized water and centrifuged at 9000 rpm for 30 min repeatedly. The resulting suspension was dialyzed against deionized water until neutrality was reached. Finally, sample was freeze-dried to give a white CNC powder.

### 2.3. Preparation of Thiuram Disulfide (TDS)

TDS was synthesized according to the previous report [49]. 2-(ethylamino)ethanol (35.6 g, 0.4 mmol) and CHCl_3_ (200 mL) were charged into a 500 mL round-bottom flask. Then, I_2_ (25.4 g) and CS_2_ (12 mL) were added slowly, stirring under ice bath for 3 h. The reaction mixture was washed with cold deionized water repeatedly to remove aminehydroiodide. The organic layer was evaporated under vacuum. The crude product was purified by a column chromatography (silica gel, [hexane]/[ethyl acetate] = 3/2, *v*/*v*) to yield a yellow oil (14.04 g, 43% yield). 1H NMR spectroscopic measurements were recorded at 25 °C on a 400 MHz Bruker instrument (BRUKE AVANCE III, Karlsruhe, Germany). ^1^H NMR (400 MHz, CDCl_3_): δ = 4.1 (multiplet, 6H, CH_2_), 3.2 (singlet, lH, OH), 1.4 (triplet, 3H, CH_3_).

### 2.4. Preparation of CNC-Containing Gels

To prepare the gels with different CNC contents, a determined amount of CNC (0 mg, 50.0 mg, 70.0 mg, 100.0 mg, 120.0 mg and 188.4 mg) was dispersed in 3.6 mL of DMF by stirring. TDS (472.8 mg, 1.44 mmol), TEA (168.5 mg, 1.13 mmol), HDI (481.6 mg, 2.87 mmol) and DBD (3 drops, 0.5% for isocynate units) were charged in sequence. After stirring for 10 min, the solution was transferred into 1 mL of injection syringe for 1 day to give cylindrical organogels with CNC content of 0%, 1.1%, 1.5%, 2.2%, 2.6%, and 4.0%, respectively.

### 2.5. Characterization

The morphology of CNC was observed using transmission electron microscopy (TEM). Two drops (about 10 μL by using micropipette) of 0.006 wt % CNC ethanol suspension were deposited on a carbon-coated copper grid and then dried under ethanol atmosphere at room temperature overnight. The sample was then observed using a FEI tecnai G2 F20 (FEI Company, Hillsboro, OR, USA) with 200 KV acceleration voltages.

The healing process of CNC-based gels was monitored by an optical microscopy (Nikon Eclipse E200, Tokyo, Japan). The CNC-containing gel was split into two parts using a blade. The detached samples were contacted together without applying additional pressure and healed by exposing to the light of the optical microscopy system with power of 6 W and sample-to-light distance of 12 cm.

The tensile performances of CNC-containing gels were tested by a tensile tester (INSTRON 3365, Norwood, MA, USA). The gels sheets were molded into dumbbell shapes (2 mm in thickness, 2 mm in width, 12 mm in length). To measure the healing efficiency, the healed samples with dumbbell shape were prepared by cutting into halves by a blade, re-contacting and exposing in the light source (10 W, distance to the samples = 10 cm) for 2 min in air. All the samples were tested at a rate of 30 mm/min at room temperature. Real time recording was used to get stress-strain curve. The healing efficiency of tensile strain (*HE*_t_) and stress (*HE*_s_) are expressed as [35]

$$HE_{t}=\frac{L_{h}}{L_{p}}\times 100\%$$

$$HE_{s}=\frac{S_{h}}{S_{p}}\times 100\%$$

where *L*_h_ and *S*_h_ are the healing strain and stress of the healed samples at the breaking point, *L*_p_ and *S*_p_ are the strain and stress of pristine samples at the breaking point, respectively.

The rheological behaviors of CNC-containing gels were performed with a stress-controlled rheometer Rotational Rheometer MARS III Haake (Thermo Scientific, Karlsruhe, Germany) with a parallel plate of 35 mm diameter. Samples with a diameter of 30 mm and a thickness of 5 mm were subjected to carry out a strain sweep test for five measurements. The value of the strain amplitude was selected as 1% to ensure that all measurements were determined within a linear viscoelastic region.

## 3. Results and Discussion

**Figure 1:**
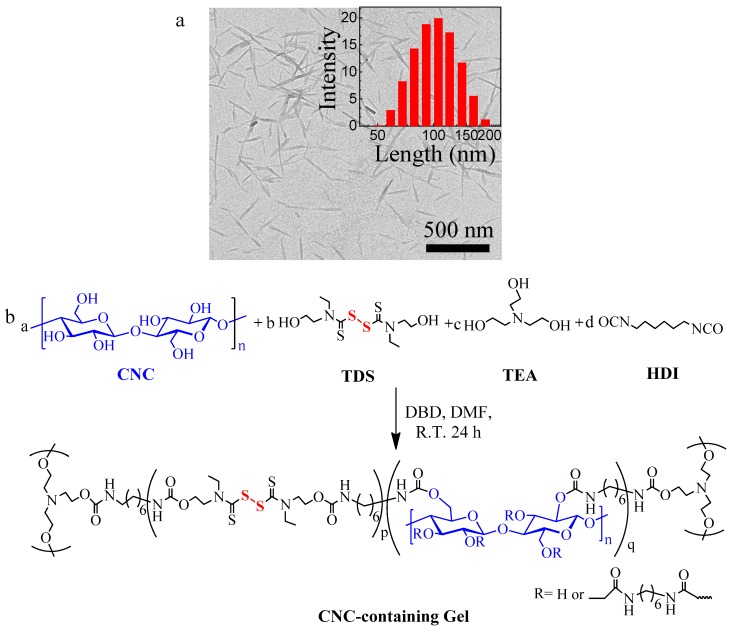
(**a**) TEM image of cellulose nanocrystals. (**b**) Schematic representation of self-healing CNC-containing gel via polyadditation reaction.

The cross-linked CNC-containing gels incorporating TDS units were synthesized via the polyaddition between CNC, TDS, and HDI. CNC were obtained from the sulfuric acid hydrolysis of bamboo pulp. The size of CNC is 199 ± 68 nm in length and 25 ± 8 nm in width, respectively (Figure 1a). Because of a large number of hydroxyl groups on the CNC surface, TDS, and TEA, the isocynate groups in HDI reacted with CNC, TDS and TEA via polyaddition to form cross-linked gels. Both TEA and CNC can be acted as crosslinkers during network formation. The dynamic TDS units with disulfide bonds which are capable of reshuffling, were incorporated in the crosslinked gel, as shown in Figure 1b. It is crucial to regulate the mole ratio of CNC, TDS, TEA and HDI to design CNC-containing gels with high self-healing efficiency and stretchable property. In our study, CNC-containing gels with a CNC content from 0% to 4.0% were fabricated.

**Figure 2:**
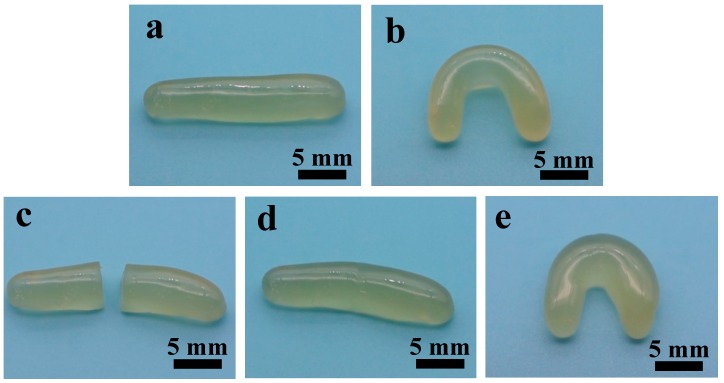
Photographs of gels with CNC content of 2.2%: (**a**) before cutting, (**b**) bending before cutting, (**c**) after cutting, (**d**) self-healing, and (**e**) bending after self-healing.

The CNC-containing gels show a remarkable self-healing ability under visible light at room temperature, without the need for applying any catalyst for healing. Figure 2a shows the gel with CNC content of 2.2% before cutting. The gel is flexible under bending (Figure 2b). For the study of self-healing behavior, the cylindrical sample of gel was cut into two pieces (Figure 2c) with a blade. After placing the two separated pieces in close contact by hand, the re-contacted samples were exposed to visible light in air for 2 min at room temperature. As shown in Figure 2d, the two pieces became a single piece essentially and no apparent boundary can be observed. Furthermore, the self-healed CNC-containing gel retained its integrity under bending without break (Figure 2e). The healing process is reversible, and the gels can experience multiple healing cycles.

**Figure 3:**
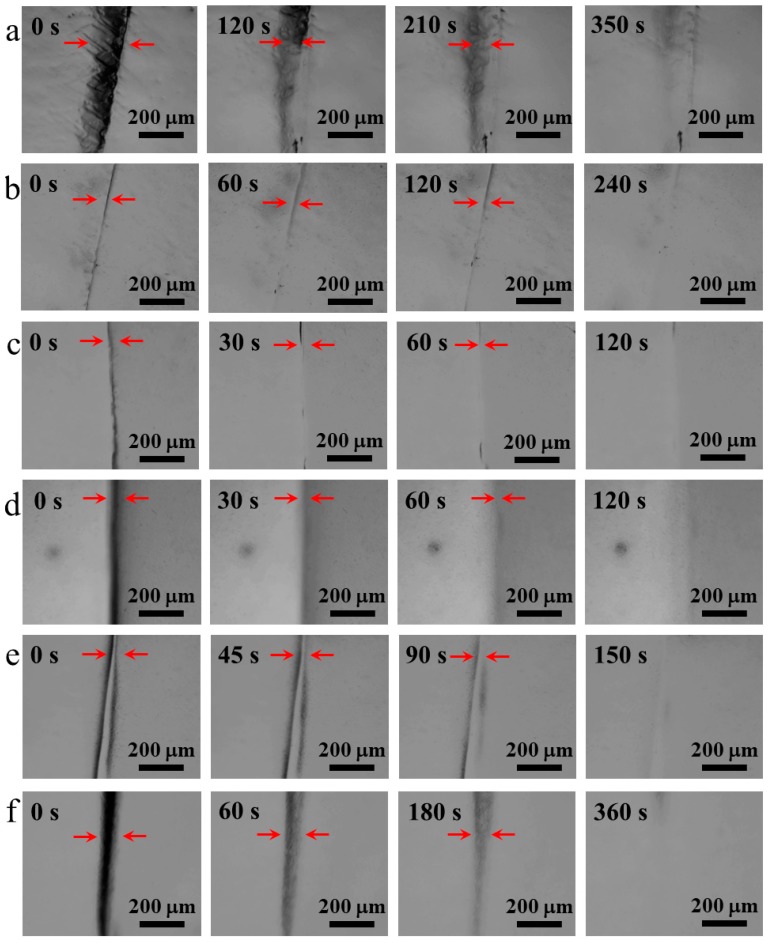
Images of gels with CNC content of (**a**) 0%, (**b**) 1.1%, and (**c**) 1.5%, (**d**) 2.2%, (**e**) 2.6%, and (**f**) 4.0% during the self-healing process (Red arrows point out cracks).

To further study the self-healing behavior of the CNC-containing gels, the self-healing process was monitored by optical microscopy. Figure 3 shows the photographic sequence of the healing process of gels with CNC content of 0%, 1.0%, 1.5%, 2.2%, 2.6%, and 4.0%. The red arrows point out the cracks during the healing process. After the gel was cut by a blade, the two separated pieces were re-contacted immediately and healed under the visible light of optical microscopy. The gels with different CNC contents show different times for healing. For the gel without CNC (CNC content of 0%), 350 s is needed to heal the sample. As the CNC content increases to 2.2%, the healing time shortens. Only 120 s is needed for the complete disappearance of the rupture when the CNC content reaches to 2.2%, showing a rapid self-healing process. However, increasing CNC concentration (2.6%, 4.0%) in the gels appear to tardy crack recovering. It is known that the aggregation of nanoparticles is unavoidable, and the number of non-dispersed particles will ascend in the CNC solution with higher concentration, which might lead to the longer healing time. To verify that disulfide bonds served as the main self-healing system via breaking and re-forming of bonds, we also measured the healing behavior of the sample in the absence of TDS. As expected, the gel with a 2.2% CNC concentration without any disulfide units, was unable to integrate when two separated samples were in contact, even for a long period (30 min).

To achieve self-healing properties, materials containing disulfide bonds usually need rigorous external stimulus to trigger the reversible reaction, such as ultraviolet light, heat, ultrasound, and electricity. It is well-known that the bond energy of disulfide is less than that of C–C, which favors the breaking and re-forming of S–S bonds. Although the exchange of aromatic disulfides is faster than the corresponding aliphatic disulfides [22], 2 h are needed to repair the crack of polyurethane prepared by aromatic disulfides [29]. However, our results showed that the gels with CNC content of 2.2% can achieve self-healing within 2 min under ambient visible light, indicating the disulfide bonds in TDS units require less energy to dissociate than aliphatic or aromatic disulfides. Thanks to the electronegativity of C=S in TDS units, the dissociation and reformation of S–S bonds are more likely to occur under mild conditions.

**Figure 4:**
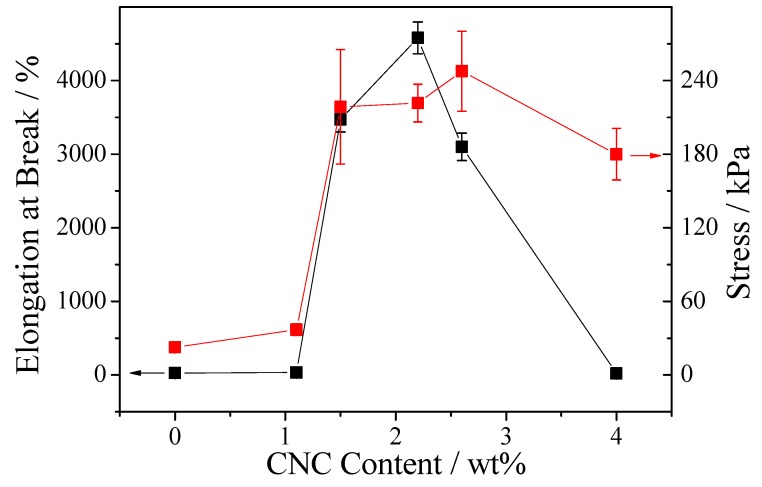
Elongation at break (black square, indicated by the black arrow) and stress (red circles, indicated by the red arrow) of CNC-containing gels.

To evaluate the mechanical property of the CNC-containing gels, tensile tests of gels with different contents of CNC were carried out. As shown in Figure 4, a similar trend that both elongation and stress increases first and then decreases can be observed with increasing CNC concentration. The gels with concentration of CNC below 1% are brittle and cannot stretch to one-fold of its original length. The gel without CNC shows the minimum stress in all the gels. As the concentration of CNC goes up, the stress of the samples increases gradually. When CNC concentration increases up to 2.2%, elongation attains to about 4260% of the original length, showing the gel is highly stretchable. The elongation is approximately 42.6 times and the stress at breaking point is ten times that without CNC. Nevertheless, it turns out there is a drop with increasing the concentration of CNC from 2.2% to 4.0%. The greater increase in CNC concentration (2.6%, 4.0%) results in a decrease in the tensile stress (4.4%, 19.4%) as well as in the fracture strain (32.3%, 99.6%) as compared to the sample with 2.2% CNC concentration. Therefore, the gels with CNC concentration of 2.2% achieved maximum strain. These results indicate that CNC significantly enhances the mechanical properties of gels containing thiuram disulfide [50]. As the CNC concentration increases, the increase in the stress of gels can be ascribed to the enhancing of mechanical strength by CNC and the energy dissipation by disulfide bonds when the gels suffer a deformation. However, high CNC concentration leads to the mechanical degradation of gels due to the formation of heterogeneous gels; homogenous gels are difficult to form because of the incomplete reaction of polyaddition at high CNC concentration [51].

**Figure 5:**
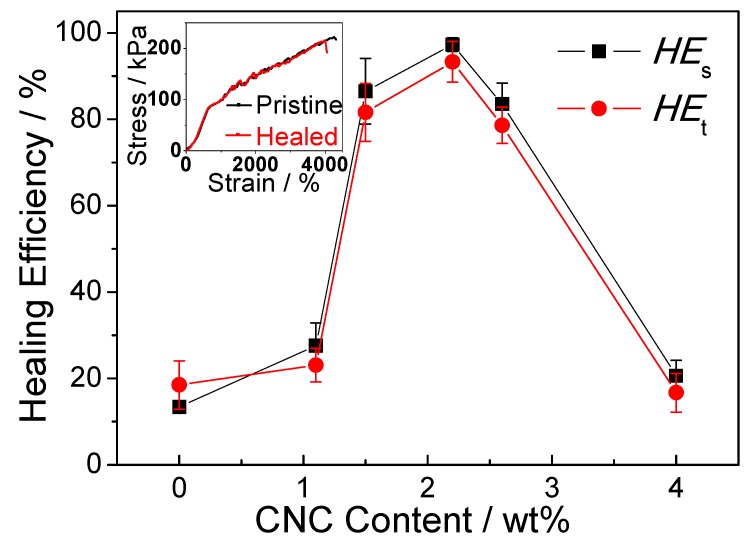
Self-healing efficiency of gels with different CNC content. Inset is the stress–strain curves of the pristine and self-healed gel with CNC content of 2.2%.

To investigate the healing behavior of cellulose-containing gels, the self-healed gel was prepared by cutting into halves by a blade, re-contacting and exposing in the visible light. The inset in Figure 5 showed the typical stress-strain curves of the pristine and self-healed gel with CNC content of 2.2%. The stress-stain curve of the healed gels was similar to that of pristine gel. The strain value of the healed gels attains to 4260%, showing that the healed gel can be stretched approximately 42 times their original length. The stress of healed gel is 215.7 ± 7.2 kPa, which is close to that of pristine sample (221.6 ± 9.0 kPa). These results indicate that the healed gels nearly restored their functionalities and structures after damage.

To better understand the healing efficiency, the *HE*_S_ and *HE*_t_ were evaluated and the calculated results were given in Figure 5. Similarly, the self-healing efficiency of samples appears to an increase along with concentration of CNC from 0% to 2.2% and decline to less than 20% when it comes to 4% concentration. The *HE*_S_ and *HE*_t_ of gels with 2.2% CNC concentration are 97.3% and 93.3% respectively, showing an excellent healing efficiency. These demonstrate that the self-healed CNC-containing gels with CNC content of 2.2% formed intact crosslinking network to withstand stress among extensive elongation. However, the lower or higher concentration of CNC cannot impart gels with high healing efficiency. This may result from inefficient chain movement and limited surface disulfide radicals exchange in brittle gels with lower or higher CNC content.

**Figure 6:**
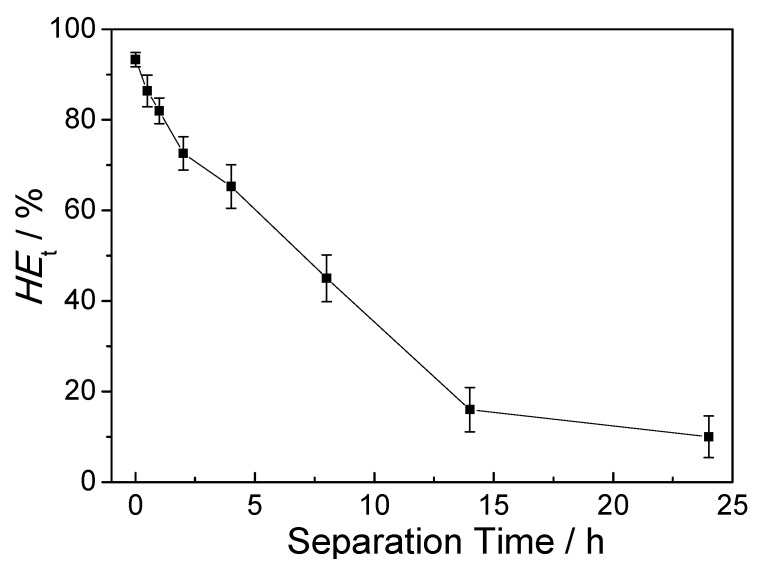
The healing efficiency of the gel for various separation times.

We further studied the influence of separation time on the healing ability of cellulose-containing gels. A gel sample made of 2.2% CNC concentration and was cut into two sections; at various times after their separation, they were then brought into contact and exposed to a visible light treatment for 2 min. As shown in Figure 6, the healing efficiency of strain decreases as the separation time increases, supporting the conclusion that the separation time after the gel was cut, is a key factor for the self-healing property; gels separated for a very long time cannot self-heal as efficiently as those newly separated. This may be caused by the reduced concentration of the thiuram disulfide bonds or radicals in the fractured surface due to the loss or migration of the TDS radicals to the interior of gel [25]. This will alleviate their accessibility to disulfides on another part when the surfaces are in contact again. Notably, the *HE*_t_ was higher than 60% for the gel separated for 4 h, indicating a high healing ability.

**Figure 7:**
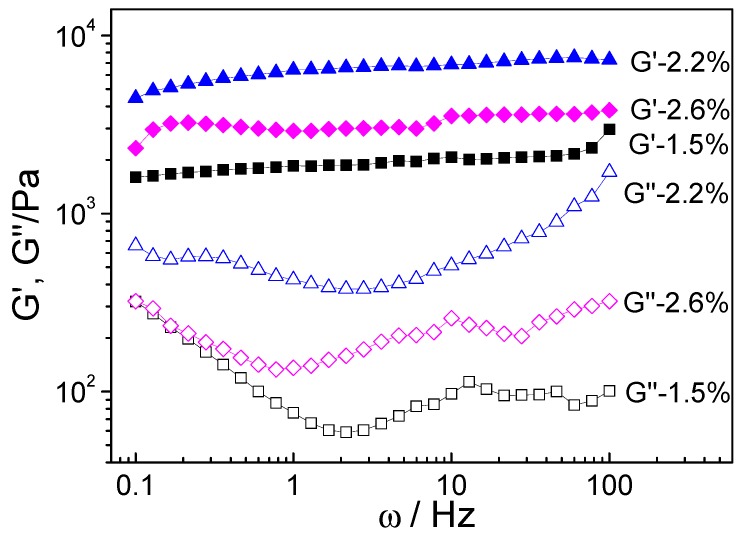
Storage modulus G′ and loss modulus G″ of the gels with CNC content of 1.5%, 2.2%, and 2.6% against angular frequency.

To gain insight into the dynamic rheological behavior, the angular frequency (ω) dependence of storage modulus (G′) and loss modulus (G″) of CNC-containing gels with CNC content of 1.5%, 2.2% and 2.6% were shown in Figure 7. It should be noted that the gels with CNC content of 0%, 1.1% and 4.0% are too fragile to perform rheology test. The G′ curves of all the gels exhibited the plateau-like behavior, indicating the rubbery plateau region of the gels network. This can be ascribed to the crosslinking structure generated by TEA and CNC. Interestingly, the G″ curve decreases at low frequency and fluctuates at high-frequency which might be due to reversible thiuram disulfide bonds in cellulose-containing gels, resulting in variations in the relaxation of polymers chains. Generally, polymer chains require significant time to rearrange themselves to minimize the free energy. It is reported that the dithiocarbamyl radicals have remarkable longevity and are stable for more than two weeks [30]. Thus, the chains have sufficient time to relax at low frequency, which result in decrease in G″ at low frequency. However, at a high frequency, the chain rearrangement might not be completed in a short timescale, leading to fluctuation in G″. In the whole frequency range, the G′ values were significantly greater than G” values, showing a gel network with elastic behavior rather than viscous nature. The G′ of gels containing 2.2% CNC (6865 kPa) shows a 332% increase in contrast to that containing 1.5% CNC (at 10 Hz), indicating that the CNC with high mechanical strength in the well-formed gels enhance the gel’s mechanical property significantly. However, further increases in the concentration of CNC leads to a decrease in G″ to 3532 kPa (at 10 Hz) for gels containing 2.6% CNC. This may be due to the brittle gel network structure caused by the higher CNC content.

**Figure 8:**
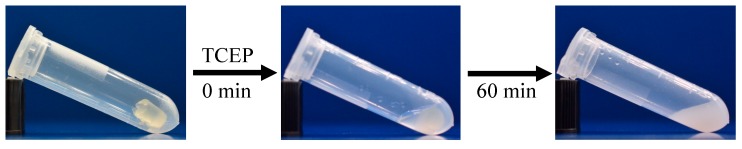
Cellulose-containing gels under reducing conditions.

To verify that the dynamic disulfide bonds control self-healing points of network structure, the gel-forming property of the cellulose-containing gels under reducing condition were investigated. As shown in Figure 8, after adding the reductant TCEP solution into the tube containing a gel, the gel was decomposed completely into a turbid solution 1 h later. Because TCEP is a strong reducing agent, the phosphine nucleophile attacked the disulfide bonds and the S-S bonds cleaved to form two thiol (-SH) groups [52]. As a result, the network of gels broke gradually, and eventually became a sol.

**Figure 9:**
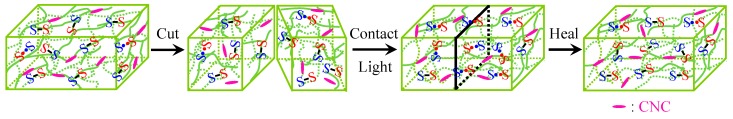
Schematic illustration of the self-healing process of CNC-containing gel.

The self-healing of CNC-containing gels is attributed to the reshuffling of thiuram disulfide bonds, as shown in Figure 9. Under the visible light, the dithiocarbamate ester bonds in the CNC-containing gels are capable of homolytic cleavage. Thus, the dormant dithiocarbamyl radical intermediates are generated, in which the unpaired electron is delocalized over the dithiocarbamate structure. After the fractured surfaces come in contact with each other, the dithiocarbamyl radicals combine to reform identical covalent S–S bonds arbitrarily across the re-contacted surface through radical crossover and degenerative radical transfer reactions [25]. Consequently, the two pieces of the organogel merged into integrity by the dynamic reversible bond. The self-healing gels fuse into their original shape after damage and restore their original properties. Therefore, the bond-shuffling reactions of thiuram disulfide enable the reorganization of the linking units in the re-contacted gel surfaces and successful self-healing of the damaged gels, which hold great potential to be applied in many application areas including engineering adhesives, sensors for radicals, vulcanizates, storage, and microelectronics.

## 4. Conclusions

In summary, self-healing cellulosed-containing gels with different CNC contents were fabricated by incorporating thiuram disulfide bonds. Via thiuram disulfide reshuffling reactions, the CNC-containing gels healed and restored their structures and mechanical properties after damage at room temperature under visible light rapidly. The thiuram disulfide-functionalized gel with a CNC content of 2.2% showed high mechanical and self-healing properties. The gel was highly stretchable and could be stretched nearly 42.6 times of their original length. This study can provide versatility in the development of self-healable CNC-containing gels with implications in many related engineering applications, such as adhesives, sensors, vulcanizates, storage, and microelectronics.
